# DHRS7 is an immune-related prognostic biomarker of KIRC and pan-cancer

**DOI:** 10.3389/fgene.2022.1015844

**Published:** 2022-10-06

**Authors:** Sheng Tang, Zhenyu Zhao, Yuhang Wang, Mariya M. El Akkawi, Zhennan Tan, Dongbin Liu, Guoxiong Chen, Hu Liu

**Affiliations:** ^1^ Department of Orthopedics, The Sixth Affiliated Hospital, School of Medicine, South China University of Technology, Foshan, Guangdong, China; ^2^ The First Affiliated Hospital, Jinan University, Guangzhou, Guangdong, China; ^3^ Department of Urology, The Sixth Affiliated Hospital, School of Medicine, South China University of Technology, Foshan, Guangdong, China; ^4^ Department of Plastic and Reconstructive Surgery, Zhujiang Hospital of Southern Medical University, Guangzhou, Guangdong, China

**Keywords:** DHRS7, pan-cancer, TCGA, KIRC, prognostic biomarker

## Abstract

Renal clear cell carcinoma (KIRC) is one malignancy whose development and prognosis have been associated with aberrant DHRS7 expression. However, the catalytic activity and pathophysiology of KIRC are poorly understood, and no sensitive tumor biomarkers have yet been discovered. In our study, we examined the significant influence of DHRS7 on the tumor microenvironment (TME) and tumor progression using an overall predictable and prognostic evaluation approach. We found novel cancer staging, particularly in KIRC, as well as potential therapeutic drugs out of 27 drug sensitivity tests. Using Perl scripts, it was possible to determine the number of somatic mutations present in 33 tumors, as well as the relative scores of 22 immune cells using CIBERSORT, the relationship between immune infiltration and differential expression using TCGA data, and the immune microenvironment score using the estimate technique. Our results show that DHRS7 is abnormally expressed in pan-cancer patients, which influences their survival. Low DHRS7 expression was associated with late clinical stages and a low survival rate in KIRC patients, suggesting a poor prognosis and course of treatment, in HNSG, MESO, and KIRC patients. We also found that DHRS7 was associated with TMB and MSI in certain tumors. Using KIRC as an example, we discovered a negative correlation between DHRS7 expression and immunological assessments, suggesting that this substance might be used as a tumor biomarker.

## 1 Introduction

DHRS7 is a member of a large family of short-chain dehydrogenases/reductases (SDR), which has at least 75 members in the human genome and is involved in a variety of physiological tasks (Persson et al., 2009; Kallberg et al., 2010). The public database of the SDR family contains about 679,000 sequences (Jörnvall et al., 2015), with extremely low sequence similarity and just a tiny number of conservative sequence regions. Alcohols, sugars, steroids, lipids, and xenobiotics are only a few of the substrate-specificities of SDRs. As a result, they carry out several cellular tasks, such as intermediate metabolic processes, detoxification, and signaling modulation (Zemanová et al., 2017). DHRS7 was first discovered in retinal epithelial cells (Haeseleer and Palczewski, 2000), but it is classified as an “orphan” SDR since little is known about its catalytic activity and physiological implications (Jacob and Jothi, 1989). The DHRS7 gene codes for two subtypes and is found on chromosome 14. Isotype 1 (38 kDa) has 339 amino acids, while isotype 2 (32 kDa) contains 289 amino acids (Stambergova et al., 2014). The human SDR enzyme DHRS7 is found in the endoplasmic reticulum membrane of the adrenal gland, prostate, gut, liver, thyroid, and other tissues. The colon, stomach, kidney, brain, and spleen are all places where it can be discovered (Keating, 1989). The mechanism of its catalytic activity, however, is still unknown.

The most frequent malignant tumor of renal cells and renal tubular epithelial cells is renal cell carcinoma (RCC). The most common subtype of RCC is renal clear cell carcinoma (KIRC) (Zhang et al., 2020). Patients with KIRC account for 80–90% of RCC patients and have a dismal prognosis (Motzer et al., 2015; Miller et al., 2019). Clinicopathologic risk variables are insufficient to identify KIRC at high risk of disease development (Majer et al., 2015). Under most circumstances, KIRC is resistant to chemotherapy and radiotherapy, and it has a greater rate of recurrence and metastasis than other RCC subtypes (Jonasch et al., 2014; Escudier et al., 2019). Although surgical resection is the most successful treatment for KIRC patients (Porta et al., 2019), 30 percent of those who have undergone surgery have developed metastases (Motzer et al., 2013). Little is known about the pathogenesis of KIRC, and no sensitive tumor biomarkers have been discovered yet (Yin et al., 2019).

## 2 Methods

### 2.1 Differential, expression analysis, and data processing

Data from RNAseq data in TPM format from TCGA and GTEx were uniformly processed in UCSC xena (https://xenabrowser.net/datapages/) using the Toil method. The TPM (transcripts per million reads) formatted RNAseq data were log2 transformed before being examined and compared. ACC; BRCA; CESC; CHOL; COAD; DLBC; ESCA; GBM; HNSC; KICH; KIRC; KIRP; LAML; LGG; LIHC; LUAD; LUSC; MESO; OV; PAAD; PCPG; PRAD; READ; SARC; SKCM; STAD; TGCT; THCA; THYM; UCEC; UCS; UVM; UCEC; U DHRS7 [ENSG00000100612] was the molecule we intended to investigate using R software (version 3.6.3) (statistical analysis and visualization), and the R package we used was primarily GGPLOT 2 [version 3.3.3.3]. (for visualization).

### 2.2 Source of mutation data

cBioPortal (http://www.cBioPortal.org/index.do) is a comprehensive open network platform that includes data mining, data integration, and visualization, and is based on the TCGA database. This website provided information on DHRS7 mutations in various cancers, including structural variation, mutation, and CNA data.

### 2.3 Relationships between DHRS7, clinical phenotype, and prognosis

Survival and clinical phenotypic data were retrieved for each sample obtained from TCGA. Overall survival (OS), disease-specific survival (DSS), disease-free interval (DFI), and progression-free interval (PFI) were utilized to evaluate the connection between DHRS7 expression and patient prognosis (PFI). The Kaplan-Meier method and the log-rank test were used to undertake survival analyses (p 0.05) of each cancer type. Survival curves were created using the R packages “survival” and “survminer.” The survival package [version 3.2-10] and the rms package [version 6.2-0] were used to create the nomogram diagram. The “ggplot2” software displays the difference in DHRS7 in each tumor’s pathological stage.

### 2.4 The link between DHRS7 expression and tumor mutation burden

The number of somatic mutations in 33 tumors was calculated using Perl scripts, and the value was corrected by dividing it by the exon length. Spearman’s approach and the “cor.test” command were used to evaluate the relationship between DHRS7 expression and TMB. The two measures were illustrated using radar plots made with the R tool “fmsb."

### 2.5 DHRS7 expression and tumor microenvironment association coefficients in cancers

The ESTIMATE algorithm was used to calculate immune and stromal scores for each tumor sample, and the relationship between DHRS7 expression and these two scores was assessed according to the degree of immune infiltration using the R software packages “estimate” and “limma.” We also used CIBERSORT, a metagene technique that may predict immunocyte phenotype, to obtain relative scores for 22 immune cells in 33 cancers.

### 2.6 The importance of DHRS7 expression in tumors from a biological perspective

Gene Set Enrichment Analysis (GSEA) was used to study TREM2’s biological activity in cancers. Gene ontology (GO) and Kyoto Encyclopedia of Genes and Genomes (KEGG) comprise gene sets, according to the official GSEA website (https://www.gsea-msigdb.org/gsea/downloads.jsp). Functional analysis was carried out using the R packages “limma,” “org.Hs.eg.db,” “clusterProfiler,” and “enrichplot.”

### 2.7 DHRS7 expression variation and clinical value in KIRC

Differential expression of DHRS7 in tumor and normal tissues was extracted using the TCGA database, which contained paired and unpaired samples. We used clinical data to map the risk variables in TCGA patients using the matching Rickscore, and to investigate the clinical relevance of DHRS7. Simultaneously, the Kaplan-Meier Plotter database (http://kmplot.com/analysis/) was utilized to investigate the DHRS7 survival curve in KIRC.

### 2.8 DHRS7 effect on biological functions, pathways, and the immune microenvironment in KIRC

In KIRC, we looked at single-gene enrichment, including Biological Process (BP), Molecular Function (MF), Cell Component (CC), and gene set enrichment (GSEA). Using TCGA data, we also looked at the relationship between the immune infiltration of 22 immune cells and DHRS7 expression, as well as the relationship between the immune microenvironment score and DHRS7 expression using the estimate technique. Meanwhile, the heat map highlighted the relationship between various immune-related indicators.

### 2.9 Tumor progression effect by DHRS7

The relationship between DHRS7 and differential expression among immune subgroups was investigated using the TISIDB database (http://cis.hku.hk/TISIDB/index.php) to investigate the correlation between TXNDC9 expression and immune or molecular subtypes of different cancer types. The researchers looked at the relationship between the degree of DHRS7 expression in KIRC and the drug sensitivity of 27 different anticancer medicines. At the same time, we looked into the link between DHRS7 and several routes.

## 3 Results

### 3.1 DHRS7 expression differences in tumor and normal tissue samples

**FIGURE 1:**
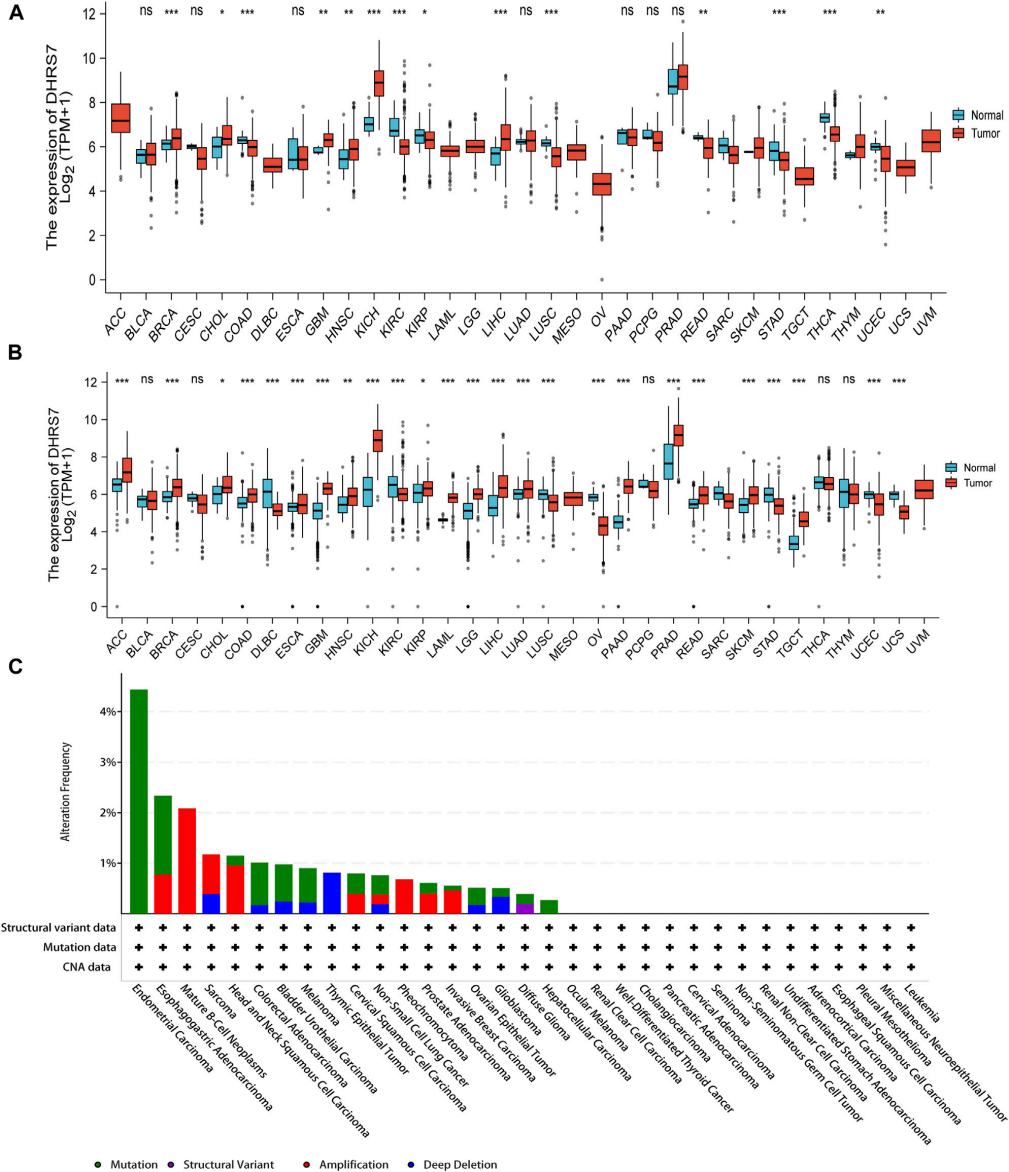
The expression level of DHRS7 gene in different tumors in multiple databases. **(A)** The gene expression of DHRS7 in different cancers in TCGA database was analyzed. *p < 0.05; **p < 0.01; ***p < 0.001. **(B)** For the absent types of cancers in TCGA, the corresponding normal tissues of the GTEx database were included as controls. The box plots were supplied. *p < 0.05. **(C)** Mutation feature of DHRS7 in different tumors of TCGA. We analyzed the mutation features of DHRS7 for the TCGA tumors using the cBioPortal tool.

We examined physiologic TREM2 gene expression levels across tissues using the TCGA data set. Tumor expression levels were higher than normal tissue in BRCA, CHOL, GBM, HNSC, KICH, and LIHC. DHRS7 levels were downregulated in tumor tissues COAD, KIRC, KIRP, LUSC, READ, STAD, THCA, UCEC as compared to normal tissues COAD, KIRC, KIRP, LUSC, READ, STAD, THCA, UCEC (Figure 1A). We used the normal data in GTEx as a supplement because many tumors in TCGA lacked equivalent normal tissue data. We discovered that the expression levels of DHRS7 in ACC, ESCA, LAML, LGG, LUAD, PAAD, PRAD, SKCM, and TGCT were considerably greater than those in normal tissues (Figure 1B). The cBioPortal database was utilized to look at DHRS7 mutation data in various malignancies, which included structural variant data, MUTATION, CNV data, and DHRS7. Endometrial Carcinoma was the tumor with the most mutations, followed by esophagogastric Adenocarcinoma, while Liver Cancer had the fewest mutations (Figure 1C).

### 3.2 The effects of four DHRS7 prognostic types in different tumors

**FIGURE 2:**
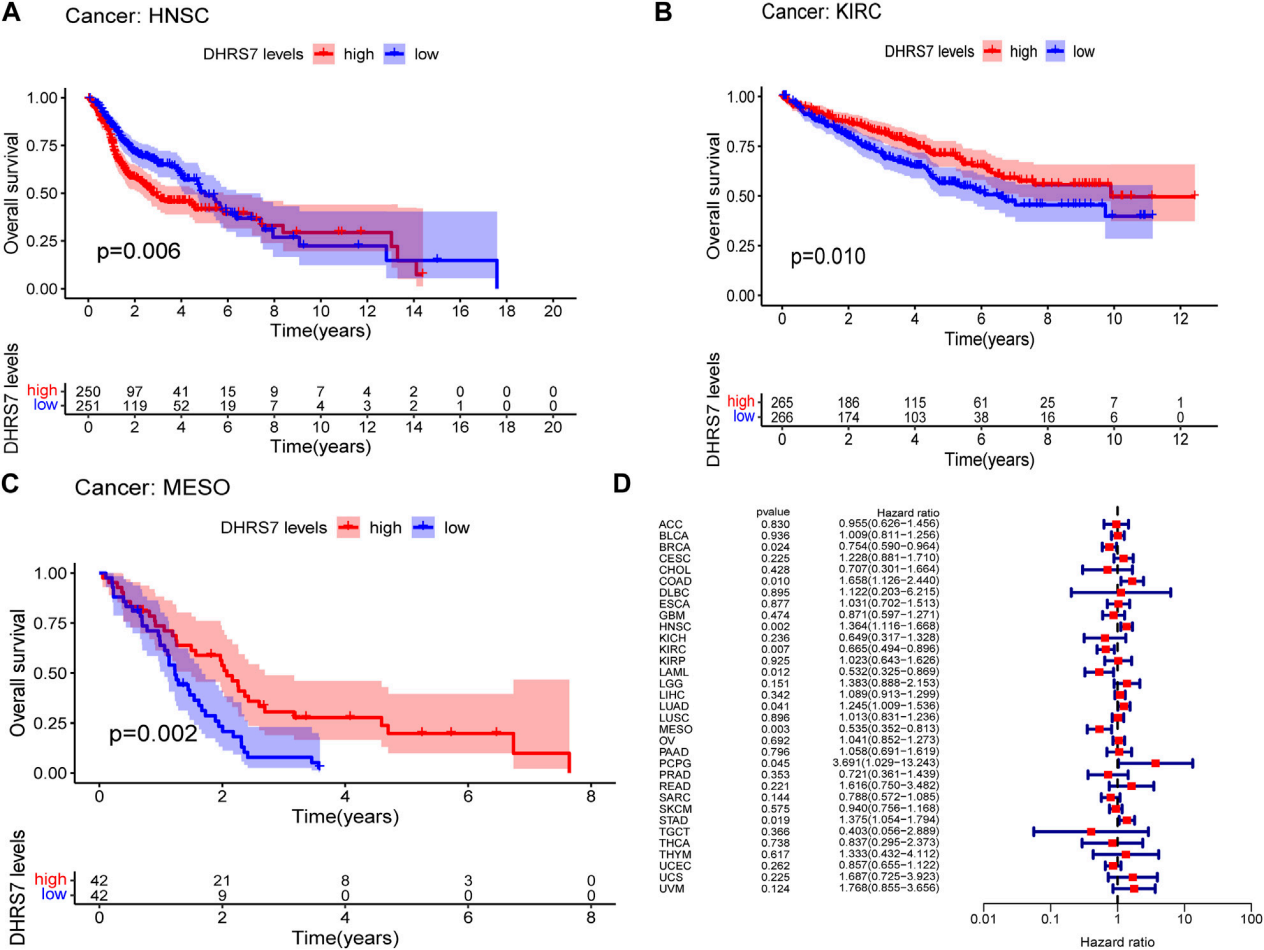
Correlation of TXNDC9 with Overall Survival in pan-cancer. **(A–C)** Kaplan-Meier curves (OS). **(D)** Forest plots showing the relationship between DHRS7 expression and OS in 33 tumor types.

**FIGURE 3:**
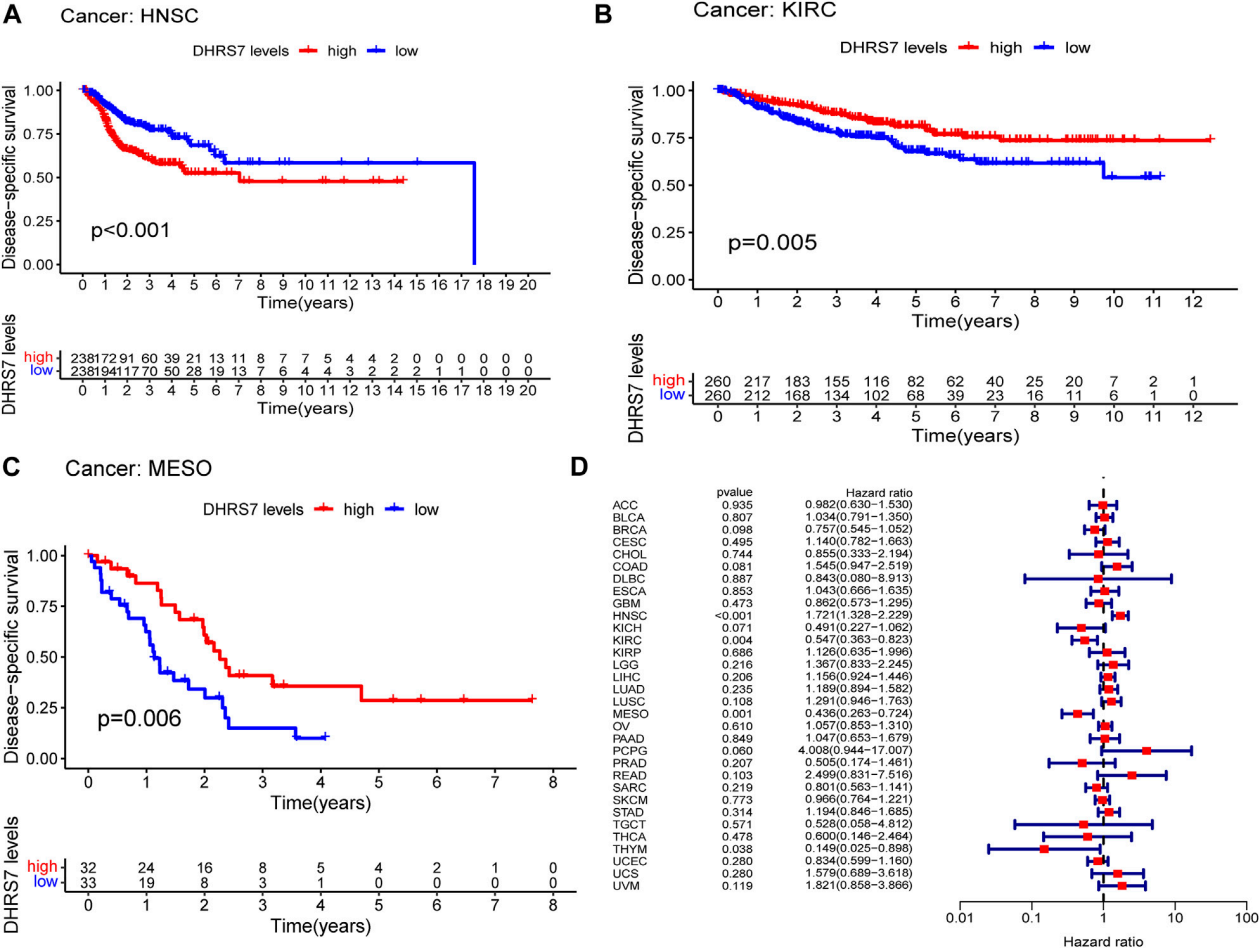
Correlation of DHRS7 with Disease-specific survival (DSS) in pan-cancer. **(A–C)** Kaplan-Meier curves (DSS). **(D)** Cox regression model analysis of the correlation between DHRS7 expression and DSS in various tumors.

**FIGURE 4:**
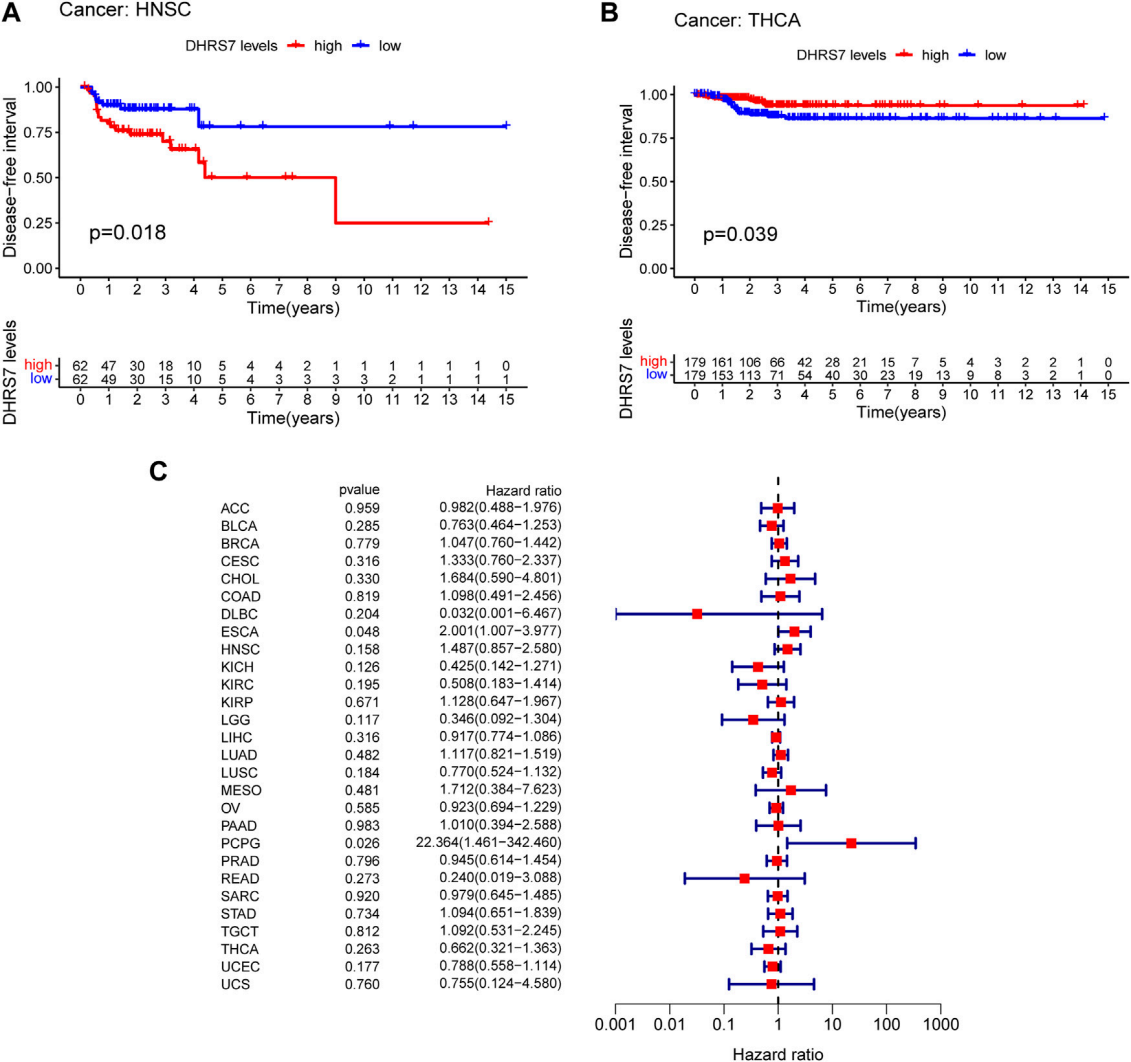
Correlation of DHRS7 with Disease-free survival (DFI) in pan-cancer. **(A,B)** Kaplan-Meier curves (DFI). **(C)** Cox regression model analysis of the correlation between DHRS7 expression and DFI in various.

**FIGURE 5:**
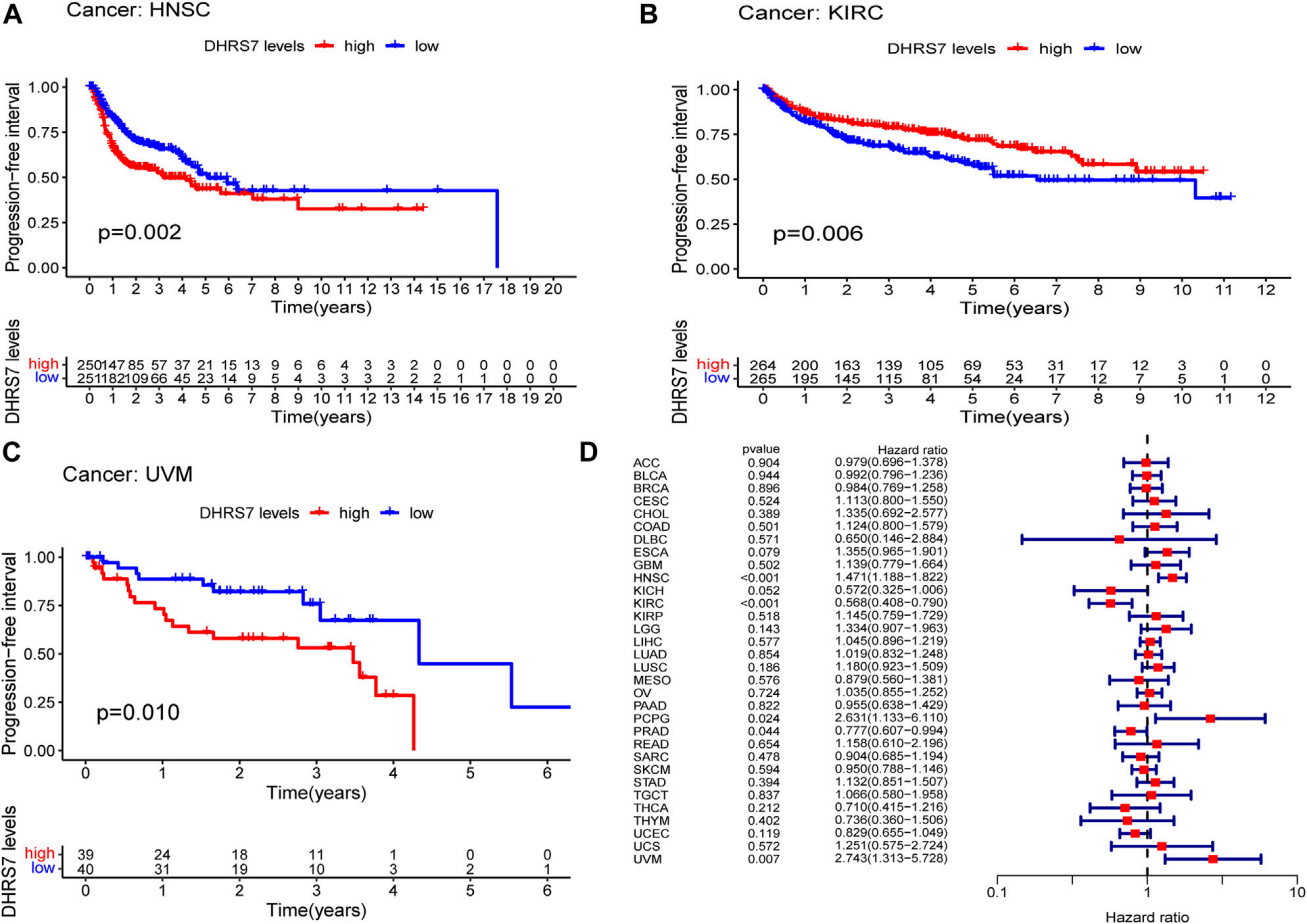
Correlation of DHRS7 with Progression-free survival (PFS) in pan-cancer. **(A–C)** Kaplan-Meier curves (PFS). **(D)** Cox regression model analysis of the correlation between DHRS7 expression and PFS in various tumors.

The low-expression group of HNSG, MESO, and KIRC tumors had a poor prognosis in terms of overall survival (Figures 2A–C). At the same time, as seen in the forest plots, DHRS7 expression was linked with BRCA, COAD, HNSC, KIRC, LAML, LUAD, MESO, PCPG, and STAD in overall survival (Figure 2D). The forest map reveals that HR of DHRS7 in HNSC, KIRC, MESO, and THYM is statistically significant; DHRS7 shows a risk factor in HNSC, but a protection factor in KIRC, MESO, and THYM; DHRS7 shows a risk factor in HNSC, but a protective factor in KIRC, MESO, and THYM; (Figure 3). The high-expression group of DHRS7 in DFI had a poor prognosis, whereas the opposite was true in THCA. DHRS7 was a statistically significant risk factor for both ESCA and PCPG, according to the forest map (Figure 4). The DHRS7 low-expression group in PFI had a poor prognosis in KIRC, but a better prognosis in HNSC and UVM. DHRS7 is a risk factor in HNSC, PCPG, and UVM, but a protective factor in KIRC and PRAD, according to the forest map. DHRS7 was shown to be low in KIRC in a previous examination of expression differences, and among numerous other prognostic types, DHRS7 indicated a poor prognosis in patients with low expression in KIRC (Figure 5). This suggests that DHRS7 will be a stable and reliable KIRC prognostic molecule.

### 3.3 DHRS7 expression and clinicopathology in pan-cancer patients

Following that, we looked into the association between DHRS7 expression and tumor stage and discovered that ASF1B expression was substantially correlated with tumor stage in seven malignancies, including KIRC, KIRP, LUSC, READ, SKCM, STAD, and THCA. We could see that the expression level of DHRS7 was lower in stage III than in stages I and II (*p* = 0.014, *p* = 0.0012) and that the expression level of stage IV was lower than that of stage II (*p* = 0.027) as the tumor advanced and decreased. As a result, we postulated that reduced DHRS7 expression was directly linked to a worse survival rate in KIRC patients. While the changes in DHRS7 expression between tumor stages were apparent, the differences between tumors of other stages were minimal (Supplementary Figure S1), and no statistically significant differences were found.

### 3.4 Correlation between DHRS7 expression and TMB or microsatellite instability in cancers

**FIGURE 6:**
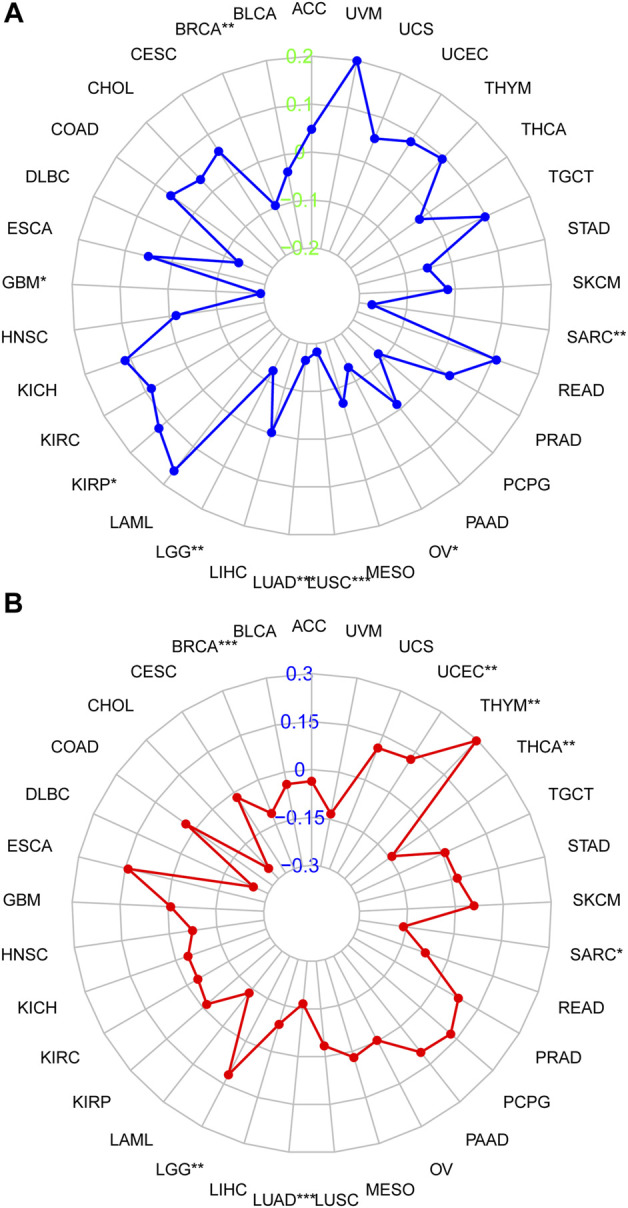
Correlation of DHRS7 expression with MSI/TMB. **(A)** Correlation between DHRS7 expression and MSI. **(B)** Correlation between DHRS7 expression and TMB.

The relationship between DHRS7 expression and TMB and MSI, which are both substantially related to ICI sensitivity across malignancies, was next investigated. DHRS7 expression was associated with TMB in a range of malignancies, according to the data. Overall, TMB expression was negatively connected with BRCA, GBM, LGG, LUAD, LUSC, OV, and SARC in 21 cancer types, but positively correlated with TMB in KIRP (Figure 6A). In addition, DHRS7 expression was found to be positively correlated with MSI in seven cancer types, including UCEC, THYM, and LGG, and negatively correlated with MSI in SARC, LUAD, BRCA, and THCA (Figure 6B).

### 3.5 TME expression and DHRS7 expression in different cancers

Many studies have demonstrated that TME plays an important role in the occurrence and progression of malignancies. The Warburg effect is a genetic change in tumor cells that results in uncontrollable proliferation, apoptosis resistance, and a metabolic shift to anaerobic glycolysis. These activities caused hypoxia, acidosis, and oxidative stress in TME, triggered ECM regulation, triggered the response of adjacent immune cells (lymphocytes and macrophages) and stromal cells (fibroblasts), aided angiogenesis, and ultimately led to cancer growth and spread. As a result, uncovering the pan-cancer relationships between DHRS7 expression and the TME is crucial. The link between DHRS7 expression and stromal and immune score in 33 cancers was investigated using the ESTIMATE method. In PAAD, PRAD, and THCA, DHRS7 expression was found to be inversely linked with stromal scores. In ACC, KIRC, PAAD, PRAD, and THCA, DHRS7 expression was found to be significantly adversely linked with immunological ratings. There are considerable distinctions between cancer kinds, according to research (Supplementary Figure S2).

### 3.6 DHRS7 expression and immune cell infiltration in cancers

The immunological prognostic value and immune association of DHRS7 were also investigated. We discovered a high negative link between DHRS7 expression and aDC, M0 macrophage, CD4 memory activated T cell, and T cells follicular helper, but a substantial positive correlation with M2 macrophage and Masting cells resting. DHRS7 expression was inversely proportional to B cells naive, plasma cells, and T cell regulation (Tregs) in HNSC, but positively proportional to Dendritic cells resting, Neutrophils, and activated NK cells. Meanwhile, in LAML, DHRS7 displayed a high positive connection with resting mast cells, but in LGG, the converse was true. All four immune cells are favorably linked with DHRS7 in lung cancer. M2 macrophages, M1 macrophages, and Tregs all have a positive correlation with DHRS7 in THCA, but aDC, Masting cells resting, and Dendritic cells resting have a negative correlation (Supplementary Figure S3). Hence, as well as DHRS7, all have a role in the formation of the immune milieu in various malignancies.

### 3.7 GSEA’s findings

The biological importance of DHRS7 expression in various tumor tissues was investigated using GESA. The results of GO functional annotation and KEGG pathway analysis are shown in Supplementary Figure S4. DHRS7 affects cell adhesion as well as many immune-related processes including apoptosis, angiogenesis, immunological responses, and immune regulation and signaling pathways, according to the findings. Some biological entries, such as GO positive regulation of cellular amide metabolic process, GO positive regulation of translation, GO regulation of sprouting angiogenesis, GO RNA polymerase binding, and GO sprouting angiogenesis, were considerably down-regulated in KIRC. The same was true of the other two renal malignancies, KICH and KIRP, in which apoptotic pathways were shown to be down-regulated. Some biochemical pathways, such as GO odorant binding, GO olfactory receptor activity, GO positive regulation of cell cycle G1 S phase transition, GO positive regulation of mitotic cell cycle G1 S phase transition, and GO sensory perception of smell, are highly up-regulated in DLBC (Supplementary Figure S4).

### 3.8 Details of clinical correlation with DHRS7 expression differences in KIRC

**FIGURE 7:**
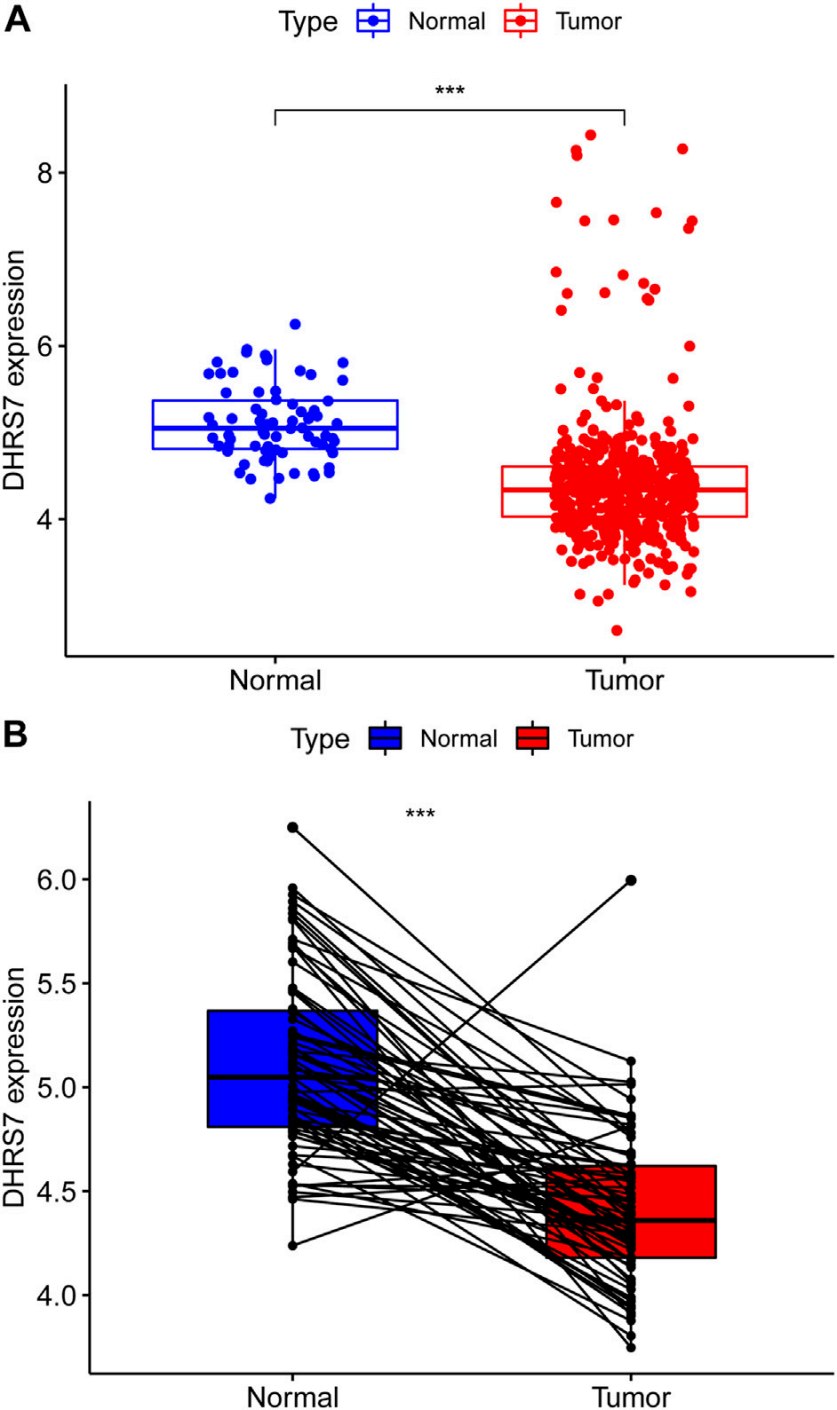
Expression of DHRS7 in renal clear cell carcinoma. **(A)** Expression of DHRS7 in KIRC in TCAG dataset. **(B)** Paired expression of DHRS7 in KIRC.

**FIGURE 8:**
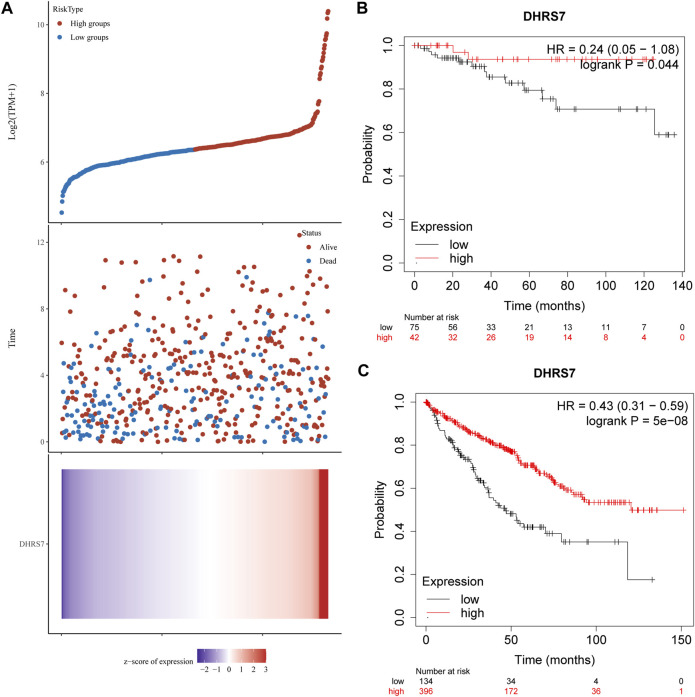
Relationship between DHRS7 expression and survival time and survival status in TCGA data. **(A)** Where the topmost scatter plot of DHRS7 expression from low to high, with different colors representing different expression groups; the middle represents the scatter plot distribution of survival time and survival status corresponding to DHRS7 expression in different samples; the bottom graph DHRS7 expression heat map. **(B,C)** Kaplan–Meier survival analysis of GXYLT2 in STAD (above: overall survival; below: progression-free survival).

**FIGURE 9:**
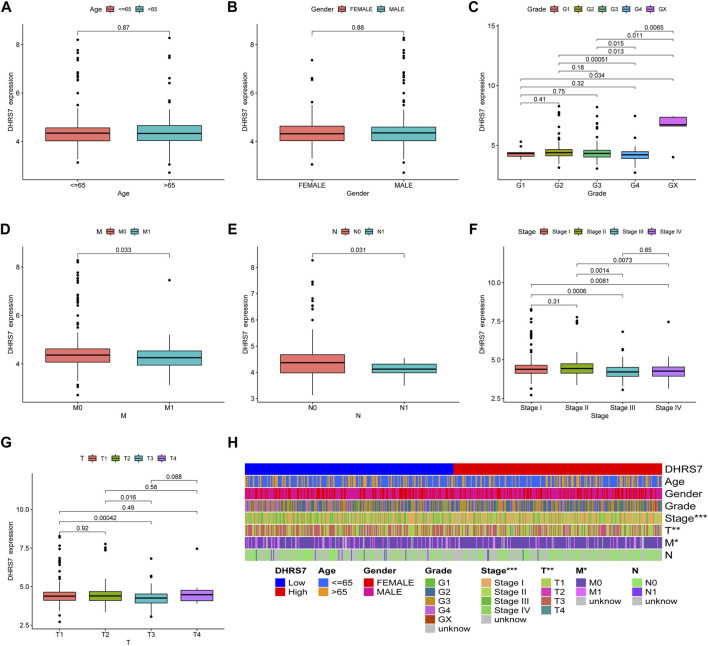
Relationship between DHRS7 expression and clinicopathology in KIRC cancer. **(A–G)** Relationship between DHRS7 expression and age, gender, primary tumor, and metastasis in KIRC. **(H)** Heatmap of age, gender, grade, stage, T, M, N in low- and high- GXYLT2 expression group.

We then conducted a more in-depth investigation of KIRC. When compared to normal tissue in KIRC, both unpaired and paired samples demonstrated lower expression of DHRS7 in tumor tissue (Figure 7). We have noticed a significant rise in the number of individuals with a Dead outcome with lower expression of DHRS7 based on the risk factor profile of DHRS7 (Figure 8A). Using the KM survival database, we also looked at the survival curves of DHRS7 in different prognostic categories of KIRC and discovered that the DHRS7 low-expression group had poor prognostic results, both in terms of OS and PFI (Figures 8B,C). More clinical correlations were investigated, and we discovered that DHRS7 expression was unaffected by age or gender (Figures 9A,B). G4 was substantially less expressed in distinct tumor grades than G2 and G3 (P<0.05), but GX was much greater than other grades (Figure 9C). Furthermore, TNM and DHRS7 have a clinical association with pathological staging. It was clear that as the tumor progressed, the expression of DHRS7 decreased, regardless of the M0&M1, N0&N1, T1&T3, T1&2, or pathological stage (Figures 9D–G). The expression levels of DHRS7 are clustered in the heat map, and both the pathological stage and TNM staging have a statistically significant clinical association, as can be shown (Figure 9H).

### 3.9 DHRS7 is required in the KIRC immune microenvironment

**FIGURE 10:**
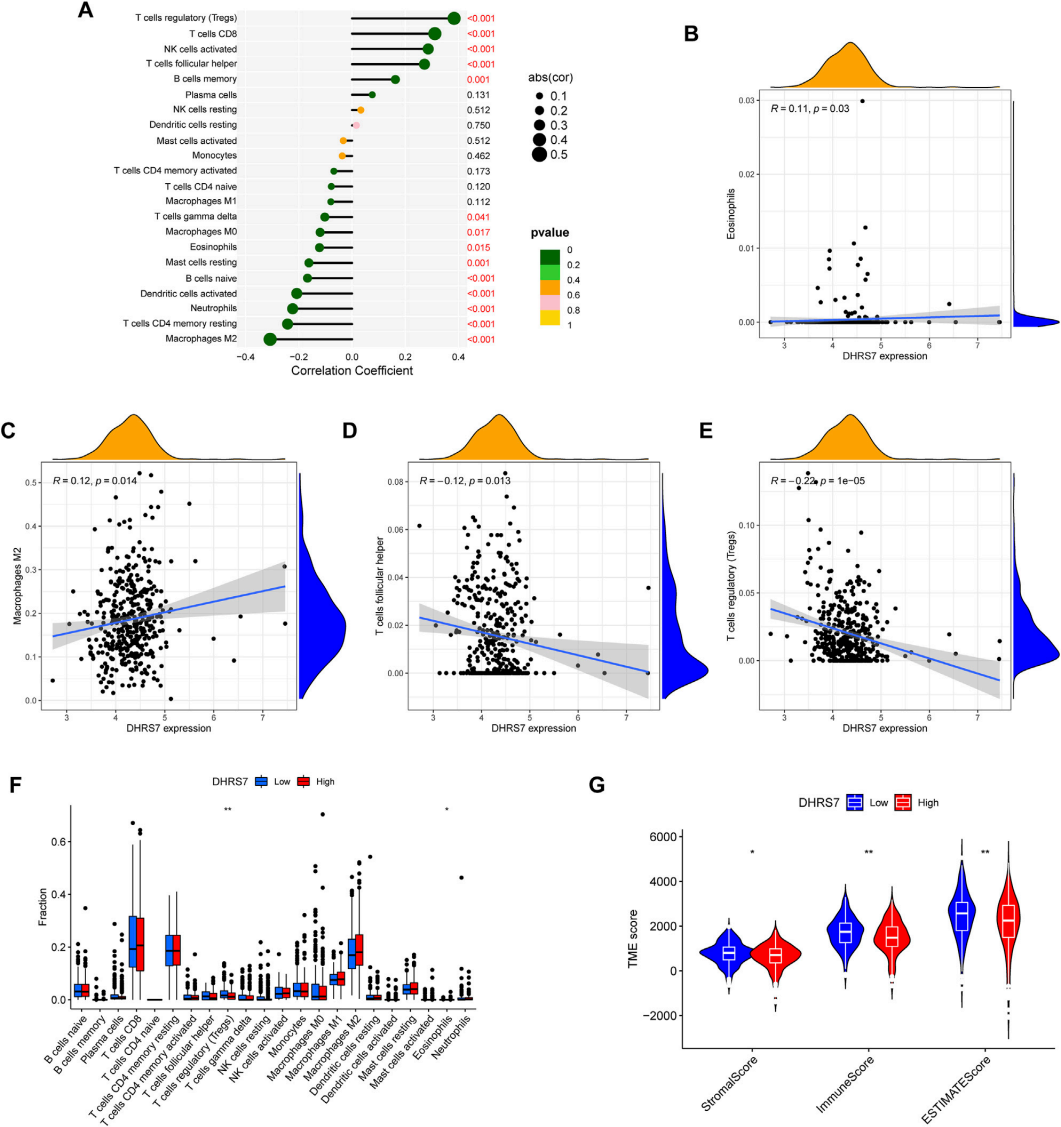
DHRS7 and immune relevance. **(A–E)** DHRS7 and immune cell correlation. **(F)** Differential expression between immune cells in high- and low-risk groups. **(G)** Differential analysis of immune microenvironment.

**FIGURE 11:**
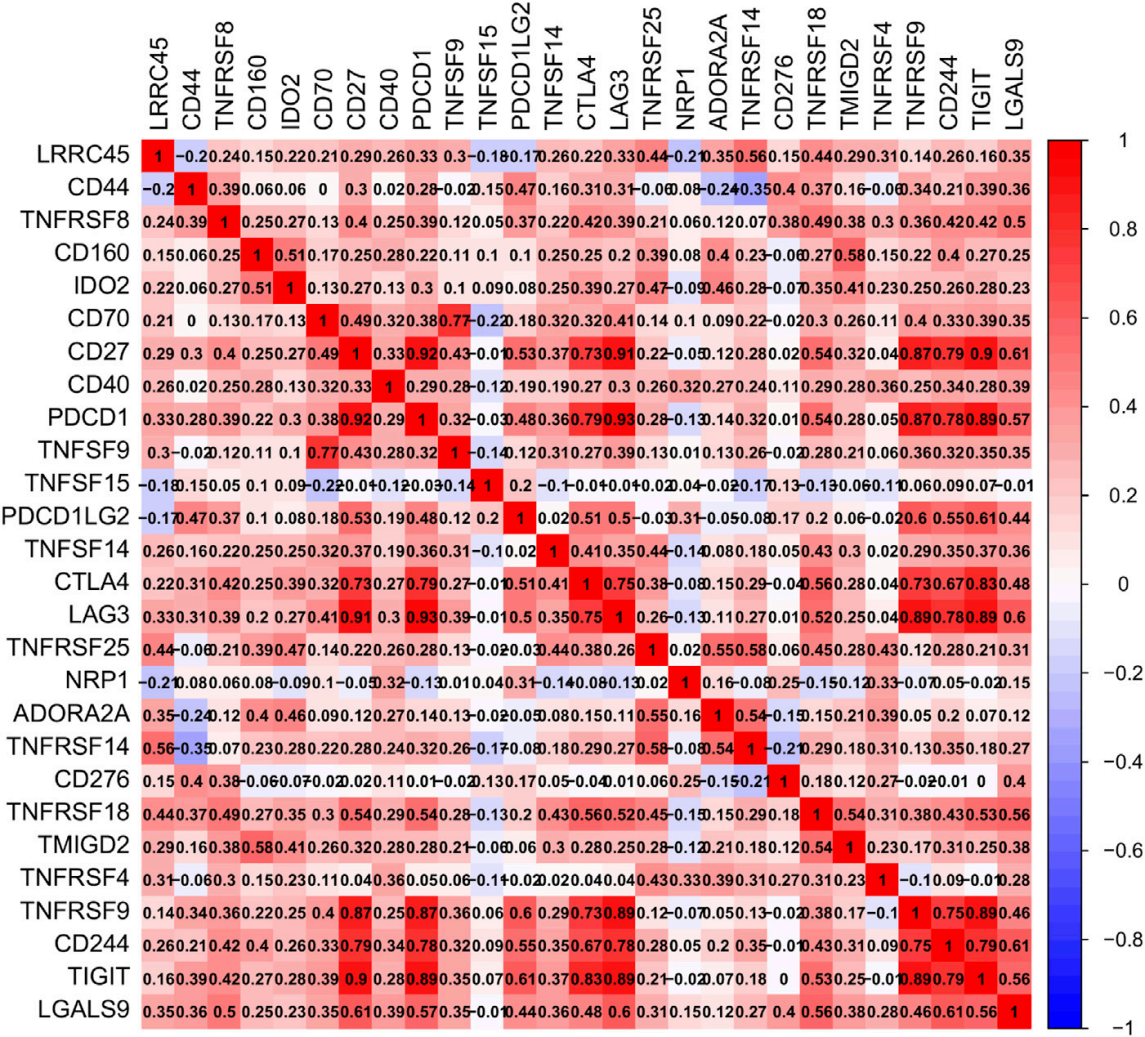
Correlation between immune genes.

Using TCGA data, we undertook an immune infiltration study to investigate the particular biological function of DHRS7 in the immunological microenvironment of KIRC. 14 of the 22 immune cells were strongly correlated with DHRS7 expression levels, as indicated in the lollipop graph. Tregs had the strongest positive association with DHRS7 expression, as in prior investigations, while M2 macrophages had the strongest negative correlation (Figure 10A). Regulatory T cells are a crucial part of immunological tolerance maintenance. They are created by the thymus and exported to the periphery, where they actively regulate the immune system by inhibiting the activation and proliferation of potential self-reactive T cells in the normal body. Many malignant disorders, such as lung, pancreas, and breast cancer, have been reported to have a considerable increase in regulatory T cells. Tumor-infiltrating macrophages (TAM) tend to polarize into the M2 type during the incidence and progression of malignancies. TAM secretes very little IL-12 after polarizing to M2, instead of producing IL-10 and TGF-, which decreases TAM’s antigen-presenting ability, inhibits T cell proliferation and killing ability, and promotes Treg and Th2 recruitment, all intending to assist tumor immune escape. When Eosinophils correlated positively with DHRS7, T cell follicular helper correlated negatively (Figures 10B–E). In the grouping comparison diagram, the Tregs infiltration score of the DHRS7 low expression group was higher than that of the DHRS7 high expression group. Furthermore, in the TME scores obtained by the estimate method, including StromalScore, ImmuneScore, and ESTIMATEScore, each TME score of the DHRS7 high expression group was significantly lower than that of the DHRS7 low expression group (Figures 10F,G). At the same time, a heat map was created to show the link between each immunological marker (Figure 11).

### 3.10 Drug sensitivity, and pathway correlation with DHRS7 expression

**FIGURE 12:**
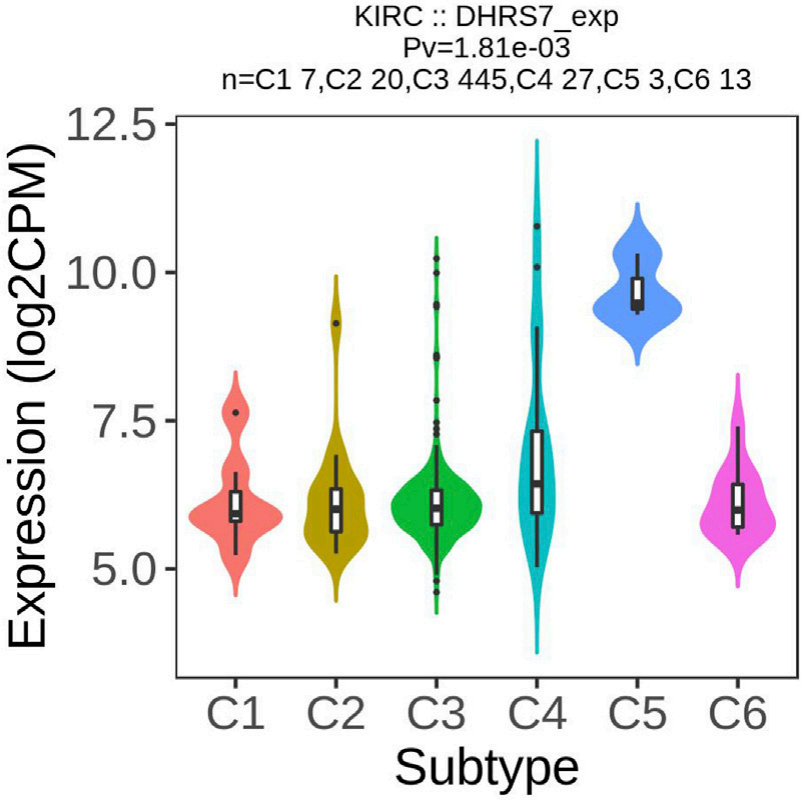
Association between KIF23 expression immune subtypes in KIRC.

We looked at the expression of DHRS7 in molecular subtypes and discovered that the expression of DHRS7 varied greatly between immune subtypes, ranging from C1 to C5, with the highest expression in C5, while the expression level of DHRS7 in C6 was equivalent to that of C3 (Figure 12). C1 (wound healing); C2 (IFN-gamma dominant); C3 (inflammatory); C4 (lymphocyte deficient); C5 (immunologically quiet); C6 (immunologically active) (TGF-b dominant). Furthermore, we investigated DHRS7’s sensitivity to 27 different medications for the treatment of KIRC. It's worth noting that the drug sensitivity in the DHRS7 low expression group was much lower than in the DHRS7 high expression group across the board (Supplementary Figure S5). Since DHRS7 is expressed at a very low level in KIRC, these guidelines suggest that decreased expression of DHRS7 will impair KIRC’s sensitivity to therapeutic medicines, resulting in a poor treatment effect and, ultimately, a poor prognosis for patients. After that, we looked into the relationship between different pathways and DHRS7 expression and found that 19 different biological pathways, including tumor inflammation signature, EMT, Cellular Response to Hypoxia, and Tumor Proliferative Signature, all had significant negative correlations with DHRS7 expression. Other immune-related pathways, such as MYC targets and Li 10 anti-infectious signaling pathway, also had significant negative correlations with DHRS7 expression (Supplementary Figure S6). All of these suggest that DHRS7 expression is lowered, which reduces immune function, enhances immune escape, and promotes tumor progression.

## 4 Discussion

DHRS7, a member of the short-chain dehydrogenase/reductase superfamily, is found in a variety of organs and tissues throughout the human body and may identify and catalyze the action of signaling chemicals such as steroids and retinoic acid (Persson et al., 2009; Štambergová et al., 2016). Previous research has shown that this enzyme can act on *in vitro* substrates (Stambergova et al., 2014), but nothing is known about how it works in the human body. Several studies have found that DHRS7 activity is reduced in prostate cancer patients (Araya et al., 2017); nonetheless, the expression of DHRS7 has been linked to cell proliferation and adhesion, suggesting that this molecule may operate as a tumor suppressor (Seibert et al., 2015). In a recent study, Diao et al. (2022) found that knocking down DHRS7 with a multi-targeted therapeutic strategy helped trigger apoptosis, indicating its potential significance in prostate cancer treatment. In the case of gastrointestinal malignancies, increased DHRS7 expression is linked to a poor prognosis in individuals with gastric cancer (Yin et al., 2021). DHRS7 is also implicated in the signaling and trafficking of cannabinoid receptors, impacting the neurological course of many malignancies, according to research (Sharaf et al., 2019). These findings suggest that DHRS7 could be used as a tumor prognostic biomarker. The TME also plays a vital role in immune evasion and treatment resistance as a significant influence linked with tumor start, development, and metastasis (Deepak et al., 2020). However, the aberrant expression of DHRS7 in cancer and the relationship between DHRS7 and TME are not well understood, which calls for more research and could lead to novel clinical targets for tumor prognosis and treatment.

KIRC is the most frequent subtype of renal cell carcinoma, accounting for over 75% of all kidney malignancies (Rini et al., 2009), with significant rates of metastasis and death (Hsieh et al., 2017). Early stages of KIPC can be treated with limited treatments such as surgical resection or physical therapy, but if it progresses, traditional medication is no longer effective, and the prognosis is poor (Hu et al., 2020). Targeted immunotherapy approaches based on gene mutation sites have increasingly been implemented in clinical practice as research on the molecular level of cancer genes has progressed. Previous research has found that the expression of many loci in KIRC is strongly linked to MSI and TMB(Liu et al., 2020; Hu et al., 2021; Zhang et al., 2021), implying that improperly expressed genes may have therapeutic potential. Meanwhile, we realized that the DHRS7-KIRC study is quite modest, which drew our attention and gave us a new perspective on cancer research.

We conducted an overall predictable and prognostic evaluation system based on the above features to analyze the strong impact of DHRS7 on TME and tumor progression, and we presented novel cancer staging, especially in KIRC, and promising medicines for clinical treatment.

In our research, we discovered that DHRS7 is abnormally expressed in pan-cancer patients and has an impact on their survival. The expression of DHRS7 was dramatically reduced in HNSG, MESO, and KIRC patients; low DHRS7 expression was directly linked to advanced clinical stages and a worse survival rate in KIRC patients, implying a poor prognosis and treatment. In addition, we found that DHRS7 was related to TMB and MSI in a variety of malignancies. Using KIRC as an example, we discovered a negative connection between DHRS7 expression and TMB, as well as immunological ratings, implying that this chemical could be used as a tumor biomarker.

We looked at the association between KIRC and TME in addition to clinicopathology and TMB perspectives. TME has an important role in the development of kidney carcinoma, according to several studies. Xiong et al. (2020), for example, looked at immune infiltration in KIRC patients and found that Tregs were down-regulated and linked with poor survival and treatment outcomes. Treg cells, on the other hand, are key regulators of inflammation and essential for immune tolerance and homeostasis (Göschl et al., 2019); Tregs are the hallmark of tumor immune infiltration and provide new therapeutic implications for cancers (Shi and Chi, 2019). Furthermore, studies have shown that M2 macrophages are linked to the KIRC tumor microbiome and can predict the prognosis of KIRC patients (Wang et al., 2021). Cancer-promoting proteins such as IL-4, IL-10, and TGF- polarize M2 macrophages, which play a vital role in anti-inflammation, angiogenesis stimulation, and tumor growth metastasis (Ngabire and Kim, 2017). Tregs had the strongest positive correlation with DHRS7 expression in our study, while M2 macrophages had a substantial negative correlation with DHRS7 expression. This shows that DHRS7 has a role in immune function and is associated with tumor occurrence and treatment. Of course, more research is needed to clarify the chemical process that connects them and corroborate our findings.

We acknowledge that our study has some limitations, given that it is a bioinformatic analysis. To begin, new animal or cell research is required to confirm our findings, as this analysis method has certain inherent flaws. Second, because the data we got from the database may be biased, some age and sex conditions that could influence the results are not taken into account. Furthermore, sample bias would be another problem we couldn’t control, however, more large-scale research could help to alleviate this issue.

Finally, abnormal DHRS7 expression has been associated with the development and prognosis of a variety of malignancies, including KIRC. As a result, we used a comprehensive evaluation model to examine the critical role of DHRS7 in pathology, clinical staging, and immune infiltration, and we confirmed that DHRS7 could be used as a biomarker for predicting tumor development and, as a result, a potential therapeutic target that needs to be investigated further.

## Data Availability

The datasets presented in this study can be found in online repositories. The names of the repository/repositories and accession number(s) can be found in the article/Supplementary Material.
