# Supplementary material for: DHRS7 is an immune-related prognostic biomarker of KIRC and pan-cancer
Source: Front Genet. 2022 Oct 6;13:1015844. doi: 10.3389/fgene.2022.1015844 (PMC9584615; doi:10.3389/fgene.2022.1015844)
Supplement: Supplementary file 1 [file DataSheet1.docx]

Supplementary Material

## Supplementary Figures


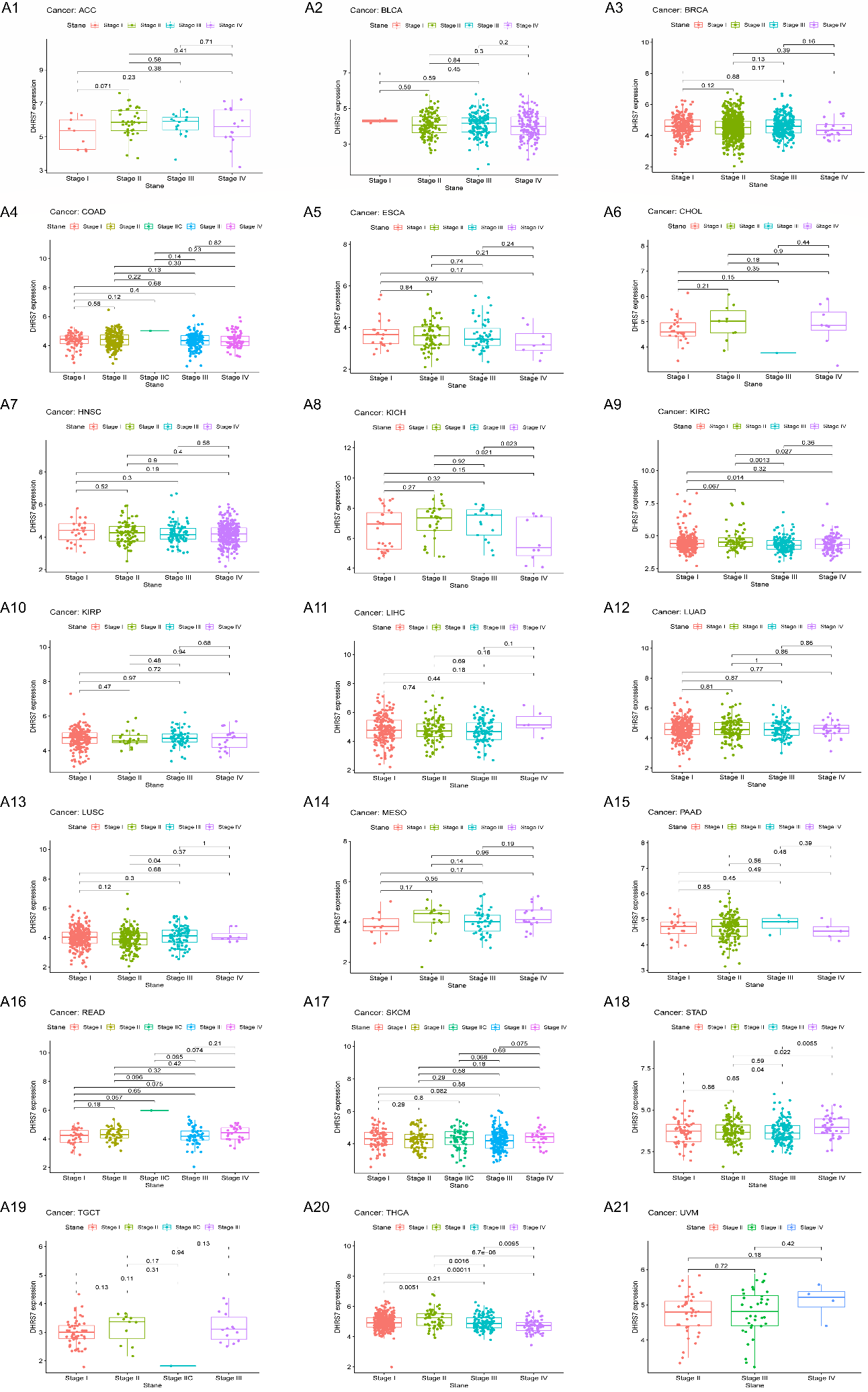


**Supplementary Figure 1.** Pan-cancer DHRS7 expression in different cancer stages


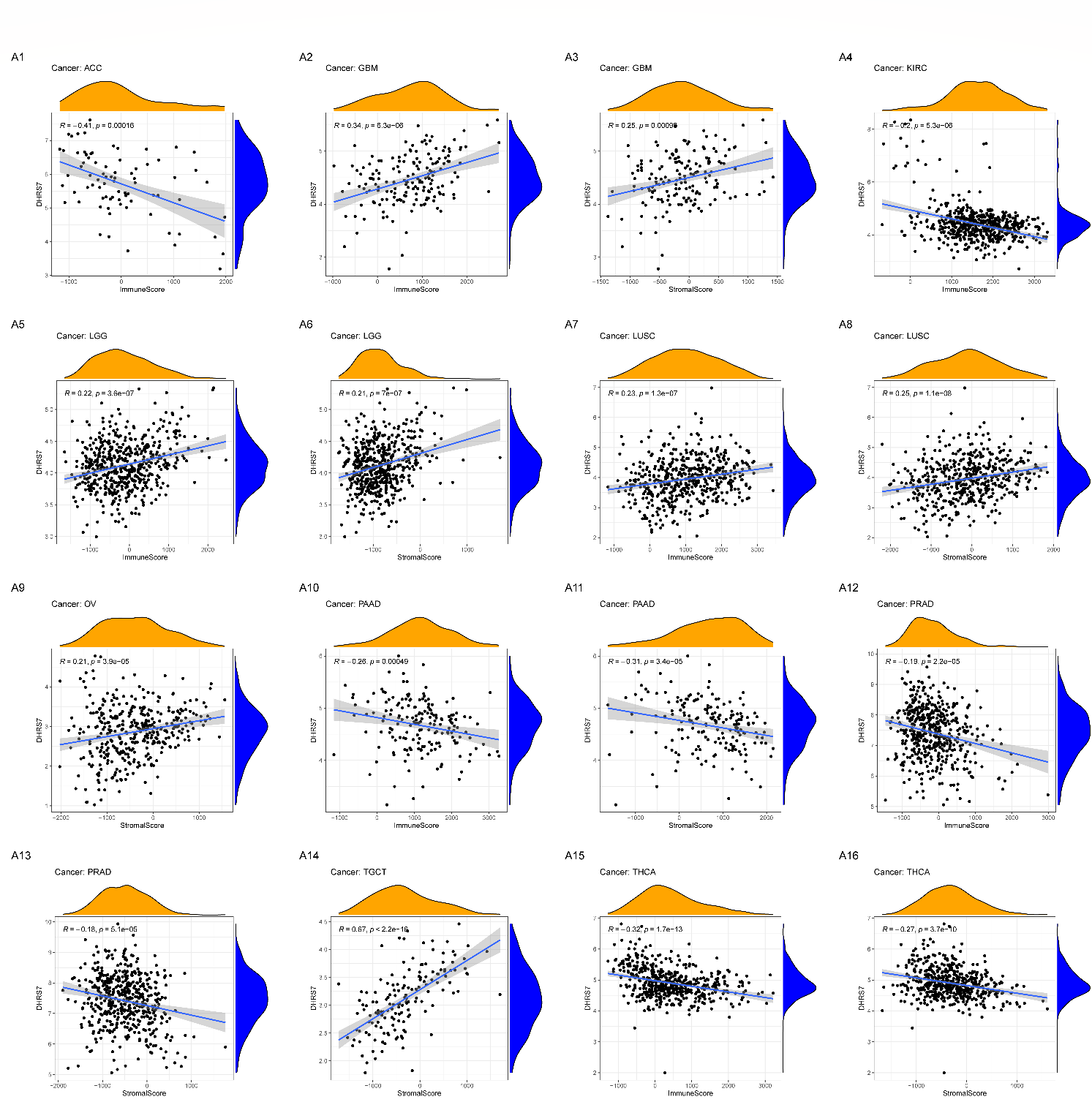


**Supplementary Figure 2** Correlation between DHRS7 expression and immuneScore and StromalScore in pan – cancer.


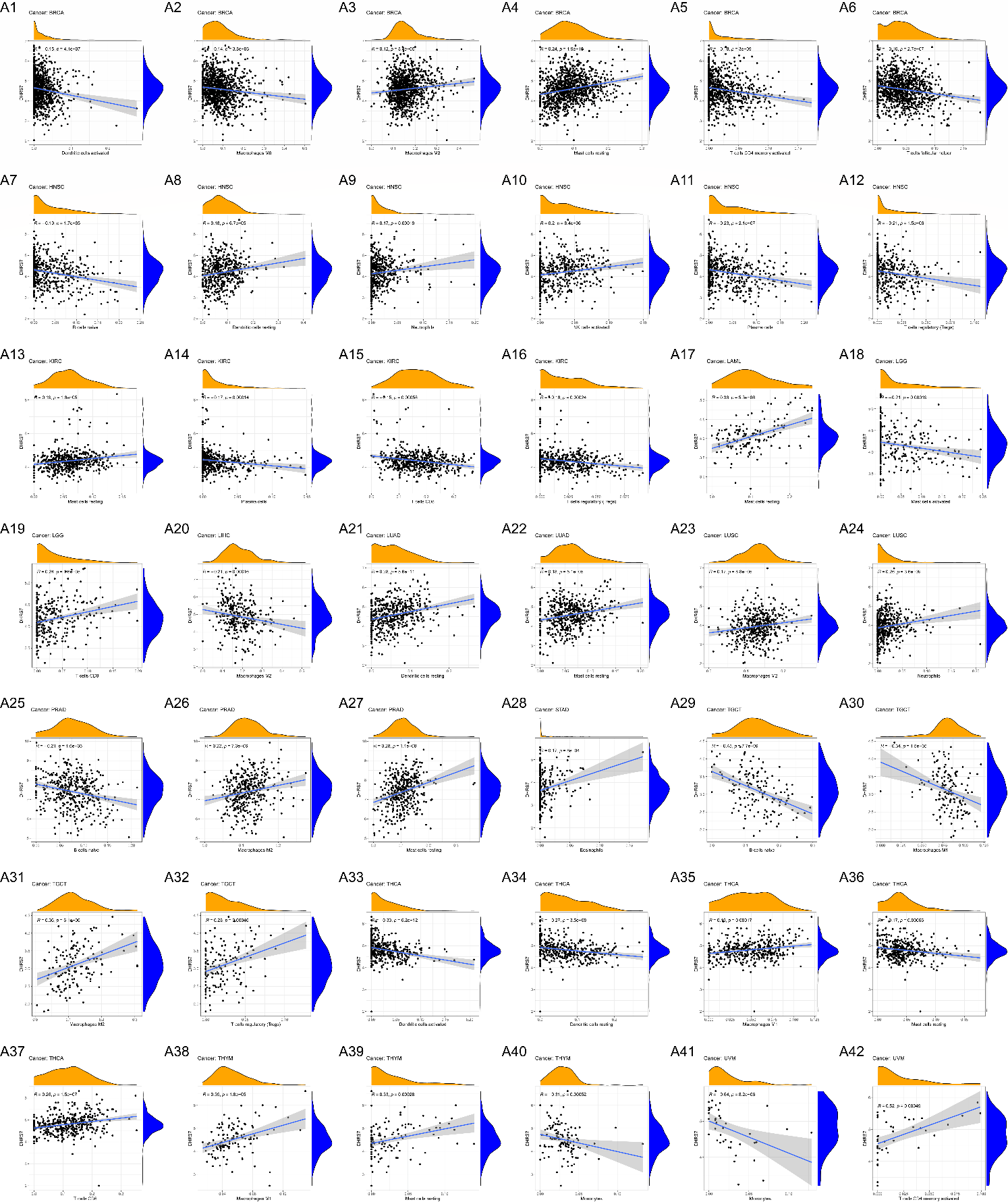


**Supplementary Figure 3** Correlation between DHRS7 expression and immunity in pan – cancer.


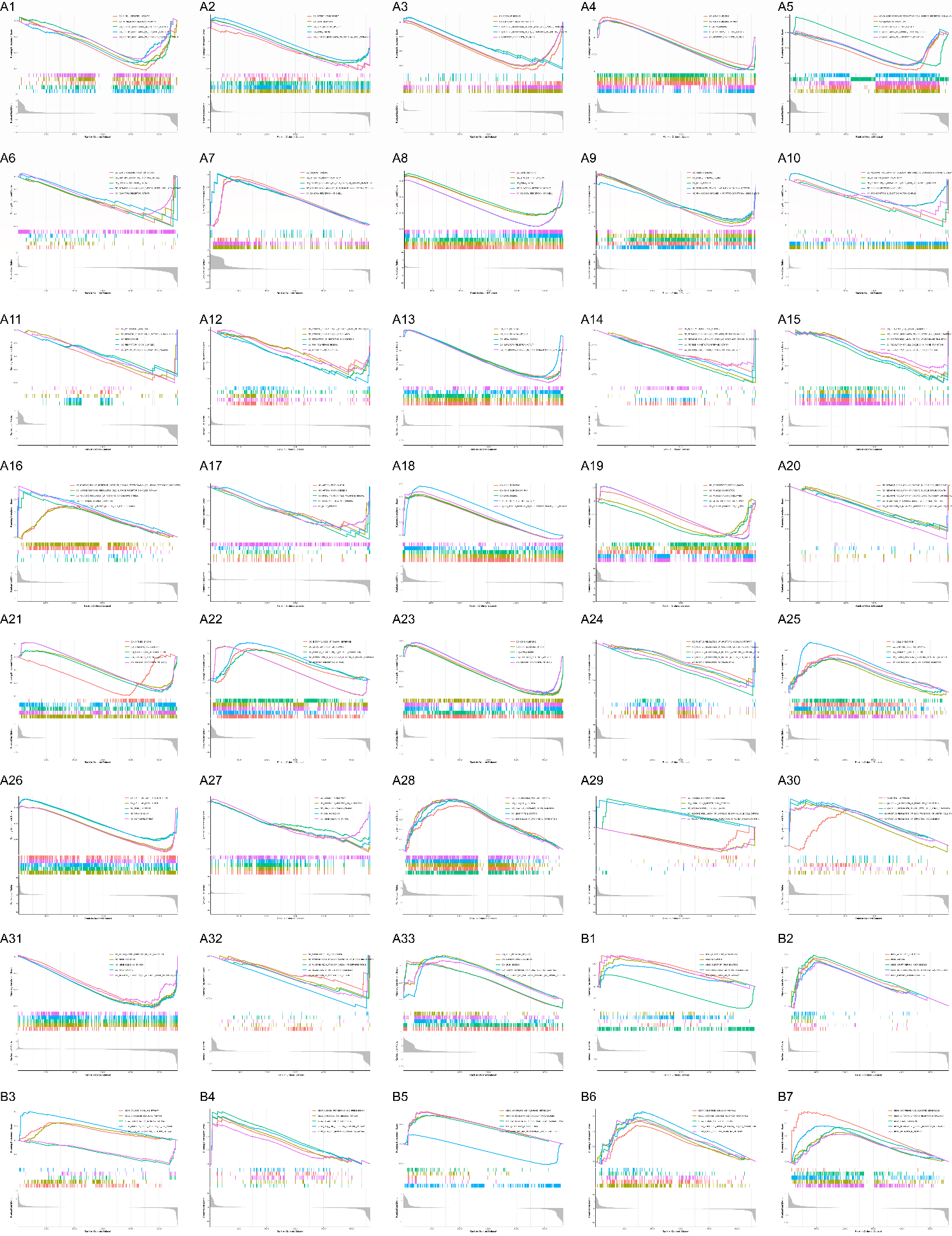


**Supplementary Figure 4** gene set enrichment analysis of DHRS7


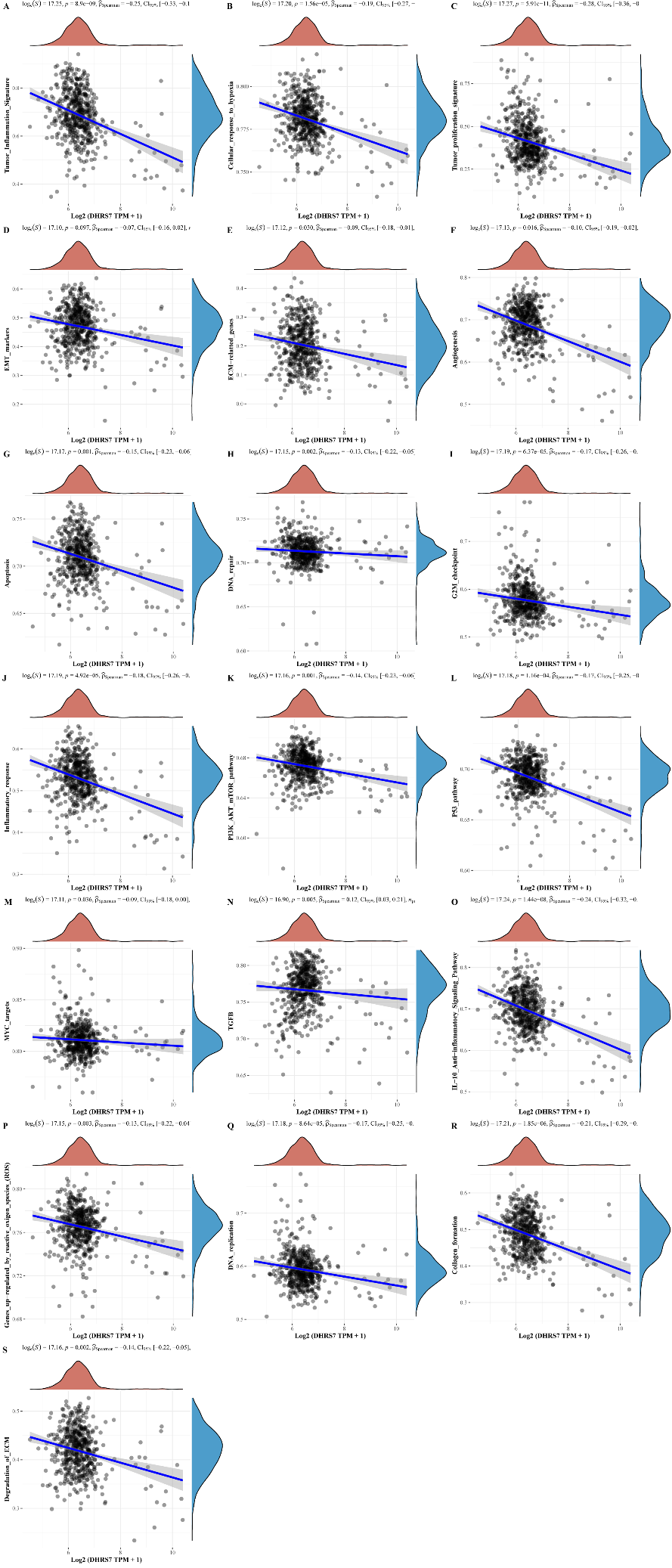


**Supplementary Figure 5** Spyarman correlation analysis between DHRS7 and pathway scores
